# Anti-HPV16 Antibody Titers Prior to an Incident Cervical HPV16/31 Infection

**DOI:** 10.3390/v13081548

**Published:** 2021-08-05

**Authors:** Ana Gradissimo, Viswanathan Shankar, Fanua Wiek, Lauren St. Peter, Yevgeniy Studentsov, Anne Nucci-Sack, Angela Diaz, Sarah Pickering, Nicolas F. Schlecht, Robert D. Burk

**Affiliations:** 1 Department of Pediatrics, Albert Einstein College of Medicine, Bronx, NY 10461, USA; ana.gradissimo@einsteinmed.org (A.G.); flora.wiek@einsteinmed.org (F.W.); lauren.stpeter@einsteinmed.org (L.S.P.); yevgeniy.studentsov@einsteinmed.org (Y.S.); 2 Department of Epidemiology & Population Health, Albert Einstein College of Medicine, Bronx, NY 10461, USA; shankar.viswanathan@einsteinmed.org (V.S.); nicolas.schlecht@roswellpark.org (N.F.S.); 3 Department of Pediatrics, Icahn School of Medicine, Mount Sinai Adolescent Health Center, Manhattan, NY 10128, USA; anne.nucci@mountsinai.org (A.N.-S.); angela.diaz@mountsinai.org (A.D.); sarah.pickering@mountsinai.org (S.P.); 4 Department of Cancer Prevention and Control, Roswell Park Comprehensive Cancer Center, Buffalo, NY 14203, USA; 5 Departments of Microbiology & Immunology, and Obstetrics, Gynecology & Women’s Health, Albert Einstein College of Medicine, Bronx, NY 10461, USA

**Keywords:** HPV16, papillomavirus, quadrivalent vaccine, antibody titer, VLP

## Abstract

The goal of this study was to investigate the serological titers of circulating antibodies against human papillomavirus (HPV) type 16 (anti-HPV16) prior to the detection of an incident HPV16 or HPV31 infection amongst vaccinated participants. Patients were selected from a prospective post-HPV vaccine longitudinal cohort at Mount Sinai Adolescent Health Center in Manhattan, NY. We performed a nested case–control study of 43 cases with incident detection of cervical HPV16 (*n* = 26) or HPV31 (*n* = 17) DNA who had completed the full set of immunizations of the quadrivalent HPV vaccine (4vHPV). Two control individuals whom had received three doses of the vaccine (HPV16/31-negative) were selected per case, matched on age at the first dose of vaccination and follow-up time in the study: a random control, and a high-risk control that was in the upper quartile of a sexual risk behavior score. We conducted an enzyme-linked immunosorbent assay (ELISA) for the detection of immunoglobulin G (IgG) antibodies specific to anti-HPV16 virus-like particles (VLPs). The results suggest that the average log antibody titers were higher among high-risk controls than the HPV16/31 incident cases and the randomly selected controls. We show a prospective association between anti-HPV16 VLP titers and the acquisition of an HPV16/31 incident infection post-receiving three doses of 4vHPV vaccine.

## 1. Introduction

Cervicovaginal infection by human papillomavirus (HPV) is the most common sexually transmitted infection in young adults. Though most HPV infections are transient, some develop into high-grade squamous intraepithelial lesions or invasive cervix cancer. HPV16 is the most carcinogenic HPV type circulating worldwide and still represents over 50% of the cervix cancers identified [1,2]. In contrast, HPV31 shares a most recent common ancestor with HPV16 and is only found in 4% of cervix cancers. Moreover, immunization with HPV vaccines targeting HPV16 cross-reacts with and can protect immunized individuals from infection with HPV31 [3].

Since HPV vaccine licensure in 2006, indisputable evidence has accumulated establishing the effective prevention of HPV infection and squamous intraepithelial lesion development [4,5,6]. Further, HPV vaccination has already demonstrated population-level vaccine impact [7,8]. For example, a recent meta-analysis of HPV vaccine programs among girls aged 13–18 years reported an 83% reduction in HPV16 and HPV18 five years post-initiation of the program [9]. As the risk of exposure persists throughout a person’s sexual life, the duration of protection, especially when the vaccine is given in the pre-adolescent period, is critical to vaccine effectiveness. For women participating in the bivalent [10], quadrivalent [11,12,13,14,15], and the nonavalent [16] HPV vaccine trials, effective protection from high-grade cervical intraepithelial neoplasia (CIN) has been demonstrated for up to 10 years among 18–26 years of age, especially among women naïve to the vaccine HPV type at vaccination. Prior reports on the immunogenicity of the HPV vaccine among children with HIV were mostly limited to 7 months post-initial vaccine dose [17,18,19,20,21]. These studies demonstrated safety and seroconversion rates above 90% amongst HIV-infected individuals, although the anti-HPV type-specific antibody’s titers were lower in the group with higher HIV viral loads [22].

Antibodies to conformational epitopes on synthetically produced virus-like particles (VLPs) are morphologically indistinguishable from authentic HPV virions and have been shown to develop in response to infection [23,24,25,26]. Serological responses directed at type-specific epitopes on VLPs are often detectable when there is clinical evidence of disease or upon receiving an HPV vaccine. Serological titers have been quantified and related to chronic infection and clinical disease status [24,27,28,29,30].

Detection of serum antibodies to HPV capsids is a valid marker for immune response prior to vaccination [31]. Since antibodies developed upon receiving the HPV vaccine are highly protective for high-grade cervical disease development, we reasoned that detection of an incident cervical HPV16 or HPV31 infection in HPV vaccine recipients would be associated with lower antibody titers compared to women similarly immunized but without detectable new infections. In this report, we investigated the serological titers of circulating anti-HPV16 antibodies prior to the detection of an incident HPV16 or HPV31 infection in a prospective post-HPV vaccine longitudinal cohort study [32].

## 2. Materials and Methods

### 2.1. Study Population

Study participants were selected from a large ongoing prospectively enrolled Phase 4 HPV vaccine cohort study of adolescent female patients attending Mount Sinai Adolescent Health Center (MSAHC) in New York City as previously described [33,34]. Study participants included sexually active patients aged 13 to 21 years at the time of enrollment who were planning to, or had received the quadrivalent HPV vaccine (4vHPV; GARDASIL^®^, Merck & Co., Inc., Kenilworth, NJ, USA) targeting HPV types 6, 11, 16, and 18. The study includes follow-up visits every six months until 25 years of age, with a collection of information by a self-reported questionnaire on lifetime and recent sexual behaviors, including the number of vaginal, anal, and oral sex partners, and a gynecological examination. Serum samples were collected at the enrollment visit, within six months of receipt of a third vaccine dose if later, and every two years thereafter for all returning participants. Additional details on the study design and protocol are described elsewhere [34,35]. The Institutional Review Board approved the study at the Icahn School of Medicine at Mount Sinai, Manhattan, New York, and written informed consent was obtained from all study participants.

### 2.2. Cases and Controls

We used a nested case–control study design. The criteria for the case groups were incident detection of cervical HPV16 (*n* = 26) or HPV31 (*n* = 17) after three doses of 4vHPV amongst baseline HPV16/31 DNA-negative individuals. Two control individuals (HPV16/31-negative) per case were selected. A random control (RC) was defined as being HPV16/31-negative throughout the study period, having received three doses of the vaccine and matched to a case on age at first dose of 4vHPV (±one year) and follow-up time. A high-risk control (HRC) was defined as a participant who was in the upper quartile of a sexual risk behavior score (score > 9), HPV16/31-negative throughout the study period, and matched to cases on age at first dose of 4vHPV (±one year) and follow-up time with complete vaccination (three doses). A linear sexual risk behavior score was derived based on the following variables, as shown in Table 1: lifetime vaginal sex partners (1–4), recent (past six months) number of partners (0–2), history of chlamydia trachomatis (0–1), any pregnancy (0–1), emergency contraception used (0–1), condom usage (0–1), ever anal sex (0–1), and lifetime number of anal sex partners (0–2). The linear score ranged from 0 and 13, and more weights were given for the number of sex partners.

### 2.3. HPV Testing

As part of the ongoing longitudinal study, detection of over 40 HPV types known to infect the cervicovaginal region was performed using a previously described protocol based on the MY09/MY11 polymerase chain reaction (PCR) in cervical Pap specimens collected at baseline (enrollment) and each follow-up clinic visit. Details on specimen collection, HPV DNA detection, and typing have been described in detail elsewhere [32,33,34,35].

### 2.4. IgG-Specific Anti-HPV16 L1 VLP-Based Enzyme-Linked Immunosorbent Assay (ELISA)

Serum samples were tested for antibodies to HPV16 VLPs as previously described [36,37]. Briefly, the HPV L1 major capsid protein was overexpressed utilizing a baculovirus expression vector in an insect cell line, resulting in VLP self-assembly [36]. VLPs were then purified by physical means and characterized for the content of L1 protein in an SDS-PAGE gel (Thermo-Fisher Scientific, Waltham, MA, USA). Transmission electron microscopy using a JEOL 1200-EX electron microscope (JEOL USA Inc., Peabody, MA, USA) was used to verify VLP assembly. A bicinchoninic acid (Pierce, Rockford, IL, USA) was used to calculate VLP protein concentration in solution [38].

The ELISA protocol was performed as described [37]. Serum samples were tested for anti-HPV16 L1 antibodies (IgG) at a starting dilution of 1:200, and then serially diluted 2-fold until 1:102,400 in 1x phosphate-buffered saline (PBS, Sigma, St. Louis, MO, USA), pH 7.4, and 0.5% polyvinyl alcohol (PVA MW 30,000–70,000, Sigma, St. Louis, MO, USA). All dilutions were tested in duplicate. Ultrapure VLPs were coated to 96-well flat-bottom PolySorp Nunc-Immuno plates (Thermo Fisher Scientific, Waltham, MA, USA) by overnight incubation at 4 °C, with 40 ng/well of anti-HPV16 VLPs. After 3 washes with 1.1x PBS (Sigma) solution with 0.05% Tween 20 (Thermo Fisher Scientific, Waltham, MA, USA), the plates were blocked at room temperature for 3 h with 1x PBS and 0.5% PVA (Sigma, St. Louis, MO, USA). After serum incubation for two hours at 37 °C and subsequent washes (1.1x PBS-0.05% Tween 20), antigen-bound immunoglobulin was detected with goat anti-human IgG (Fcγ fragment specific) conjugated with HRP (Jackson Immunoresearch Laboratories, Inc., West Grove, PA, USA) at a dilution of 1:10,000 in 1x PBS, 0.5% PVA, 0.025% Tween 20, and 0.8% polyvinylpyrrolidone (PVP360 MA 360 000; Sigma, St. Louis, MO, USA). After incubation for 1 h at 37 °C and washes, KPL ABTS peroxidase substrate (Kirkegaard & Perry Laboratories, Inc., Gaithersburg, MD, USA) was added, and color development was initiated. The reaction was stopped after 25 min by adding 1% SDS (Kirkegaard & Perry Laboratories, Inc., Gaithersburg, MD, USA), and absorbance was measured at 405 nm with a reference wavelength of 490 nm in an automated microtiter plate reader (Molecular Devices, Menlo Park, CA, USA). Seropositivity cutoff was defined at OD = 0.2 as described elsewhere [25,37,39]. Each plate tested included a known strong positive sample, a weakly reactive sample, a negative sample, and a “no sample” control. The coefficient of variation across all samples was 1–19%.

### 2.5. Serum Samples

Serum samples from cases were tested at three time points: (1) blood draw immediately prior to the visit in which the incident detection of cervical HPV16/31 occurred (pre-infection, *n* = 43; average time since the last dose of 4vHPV 3.0 ± 2.9 (standard deviation, SD) years); (2) at the time of incident detection of cervical HPV16/31 (peri-infection, *n* = 10; average time since the last dose of 4vHPV of 4.4 ± 2.9 years); and (3) the serum obtained after HPV16/31 incident detection (post-infection, *n* = 32; average time since the last dose of 4vHPV of 6.5 ± 3.0 years). Serum samples from controls were matched by time-to-case pre-infection samples (*n* = 86; average time since last dose of 4vHPV of random and high-risk controls were 2.7 ± 2.3 years and 3.2 ± 2.3 years, respectively).

### 2.6. Statistical Analysis

Descriptive statistics were used to summarize adolescent and youth characteristics. Continuous scale variables were summarized using mean and standard deviation; non-normal variables were log-transformed. The difference in continuous variables was assessed using analysis of variance and *t*-tests. The skewed data were evaluated using a nonparametric Kruskal–Wallis test. Categorical variables were presented as frequency counts and percentages, and associations were examined using the Cochran–Mantel–Haenszel modified ridit score test. The association between exposure serum titer values (log-transformed) and case–control status (incident HPV16/31) was investigated using conditional logistic regression. A logistic regression model was fitted to assess the log serum titers’ association with HPV infection under subgroup analysis.

An analysis examining the influence of infection on log serum titers post-vaccination among cases was performed. For this analysis, the changes in log serum titer before and after infection were fit using a piecewise linear mixed-effects model with random intercept and slope. Initially, actual time was computed as the difference between the last vaccination (dose 3) and pre-, peri-, and post-infection dates. Further, (relative) time for the model was recoded as time since infection and could be positive or negative. The estimation was performed using a restricted maximum likelihood approach with Kenward–Roger degrees of freedom adjustment. This model allowed for the modeling of inherently unbalanced repeated measures data and missing information under “missing at random” assumptions. All analyses were performed using SAS software version 9.4 (SAS Institute, Cary, NC, USA).

## 3. Results

The characteristics of the participants in this nested case–control study are presented in Table 1. Overall, 55 (3.9%, 95% CI: 3.0–5.1) adolescents had HPV16 or HPV31 incident infections in the MSAHC HPV study cohort (*n* = 1398) through May 2021, of whom 43 (3.1%; 95% CI: 2.2–4.1) developed infection after having completed the full set of immunizations (i.e., three doses of 4vHPV). The selected individuals (43 cases and 86 controls) were 18 ± 1.3 (SD) years of age, and 95% reported being either Hispanic or African American. Over 62% (81/129) had more than three lifetime sexual partners. The average ages at coitarche and receiving the first vaccine dose were 14.7 ± 1.4 (SD) and 15.2 ± 2.3 (SD) years, respectively. Slightly more than one-third (38%) initiated sexual activity after they had at least one dose of vaccination. We selected two different control groups to compare women with incident HPV16 or HPV31 infections. Specifically, we included a random control group (RC) that was matched on age at first dose of 4vHPV, having completed three doses of HPV vaccine and being HPV-negative for HPV16 and 31. We selected a second control group (HRC), reasoning that women with high risk for a cervical HPV infection should have higher antibody levels, affording greater protection consistent with no detectable HPV16 or HPV31 infections after immunization.

### 3.1. Comparison between Cases and Controls

Titration curves of anti-HPV16 VLP antibody titers were determined for all cases and controls in duplicate. As previously described, a serological titer equal to 0.2 OD units was defined as the seropositivity cutoff value [40]. Mean serological titers by study group are displayed in Table 1.

**Figure 1:**
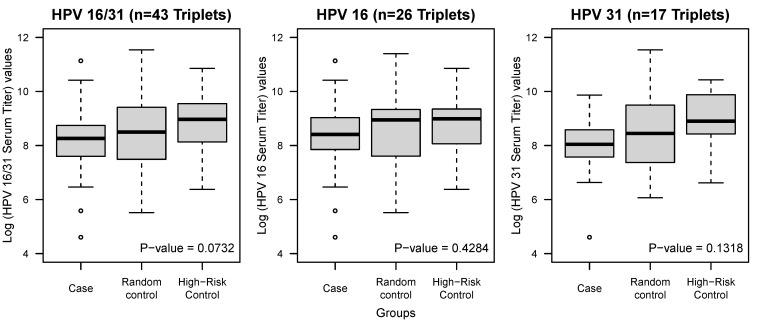
Distribution of log titer values according to the case–control group. Boxplots illustrate the distribution of log titer values by group as defined below each panel: (**Left**), HPV16 and HPV31 incident cases with matching controls (each group *n* = 43); (**Middle**), HPV16 incident cases only with matching controls (each group *n* = 26); (**Right**), HPV31 incident cases only with matching controls (each group *n* = 17). Cases and controls were tested using ANOVA, and the *p*-value is shown at the bottom right corner of each panel.

The average estimated log serum titer values among cases, random controls, and high-risk controls were 8.2, 8.5, and 8.8, respectively. The larger the log values, the higher the anti-HPV16 IgG antibody titer, allowing detection with VLP antigen at a greater dilution. The difference between the titers was marginally significant at the *p* = 0.07 level among the groups using the analysis of variance procedure. Compared to each control separately, the difference between case titers was significant with high-risk controls (*p* = 0.01) but not with random controls (*p* = 0.19) (Figure 1).

Since many risk factors need to be considered in predicting the risk of a vaccinated individual acquiring an HPV infection, we used conditional logistic regression analysis to adjust for having initiated sexual activity before taking the 4vHPV. The results indicated that, on average, the controls (RC and HRC) had a 41% higher likelihood (relative odds) of having a one log unit increase in antibody titers compared to the cases (OR = 1.41, 95% CI: 1.01–1.96; *p* = 0.04), adjusting for the receipt of the 4vHPV before sexual coitarche vs. not, as shown in Table 2. This translated to cases being 29% less likely to have 10-fold lower antibody titer compared to matched controls. When modeled individually and comparing each type of control to the HPV16/31 incident case, the results suggested that the high-risk controls had a 73% higher likelihood (OR = 1.73, 95% CI: 1.03–2.88; *p* = 0.04) of having a one-unit increase in log antibody titers. However, the relative odds (OR = 1.25, 95% CI: 0.88–1.79) were attenuated and were not significant (*p* = 0.21) compared to the random control group. Thus, high-risk controls had higher antibody titers compared to random controls and cases. A subgroup analysis of HPV16 or HPV31 types suggested similar scenarios with higher odds among the controls than cases for one log unit increase in antibody titers. This observation was more pronounced among HPV31 incident cases, where we observed the odds ratio was 67% (OR = 1.67, 95% CI: 0.96–2.92; *p* = 0.07) higher among the controls when compared to cases (Table 2).

We also assessed the association between log serum titers and HPV16/31 status, restricting to participants who completed vaccination before initiating sexual activity (Table 3). For this analysis, we unmatched the case–control pairs and assessed the association using logistic regression. The results suggest that those who were vaccinated before coitarche had higher odds of one log serum titer increase among the controls than the cases, meaning that the cases were 32% less likely to have higher antibody titers than controls.

### 3.2. Subgroup Analysis: A Longitudinal Evaluation

We also examined the changes in log serum titers before and after infection among the cases; the average log titers remained relatively stable over time in the three visits evaluated (Table 4). The average times to pre-, peri-, and post-infection serum collection from the last vaccine dose were approximately 3.0, 4.4, and 6.5 years, respectively. The relative serum collection time (pre- and post-) from infection was approximately 1.4 and 1.5 years, respectively.

Based on the piecewise linear mixed model, the average log serum titers increased from pre- to peri-infection by 0.16 units (standard error, SE: 0.14), although this was not statistically significant (*p* = 0.25), while the average log serum titers decreased by 0.11 units (SE: 0.13) from peri- to post-infection (*p* = 0.41). This case analysis suggests that potential “false negative” undetected HPV infections were not driving the higher antibody titers detected in the high-risk control group.

## 4. Discussion

We performed a nested case–control study to investigate whether antibody titers to the 4vHPV vaccine might be associated with the acquisition of an incident HPV16 or HPV31 infection, since the vaccine is expected to protect from these infections. The results suggest that the average log titers were higher among high-risk controls than the incident HPV16/31 cases and the randomly selected controls. The random control titers were also higher than the cases, although the difference was not significant. All immunized females tested in this study had detectable anti-HPV16 IgG antibodies. It is unclear whether such high titers present in the high-risk control group (HRC) prevented the acquisition of new HPV16/31 infections, or if continuous or frequent exposure (through sexual activity) induced the higher titers detected in the high-risk control group.

Though neutralization assays are often considered the “gold standard” in assessing the protective immunogenicity of prophylactic vaccines, they are highly correlated to ELISA titers. Thus, the anti-HPV16 VLP ELISA represents a surrogate assay for neutralizing anti-HPV IgG antibodies [25,37]. Additionally, the phylogenetic relationship shared by HPV31 and HPV16 translates into cross-protective humoral immunity for HPV31 from the HPV16 antigens [29,40]. In a follow-up study with unvaccinated sexually active college females [29] in which the serological evidence of protection for the acquisition of new HPV infections was assessed, the results showed that women with high levels of IgG to HPV16 VLPs in two or more preceding visits had a lower risk for subsequent HPV infection (i.e., alpha9 HPV16-related types) than those without (relative risk = 0.49; *p* = 0.037). The results included a phylogenetic group approach of different HPV types, in which the presence of prior anti-HPV16 antibodies were markers for immunity against HPV types other than HPV16. Based on the cross-protection of HPV31 by HPV16 vaccination [23,41,42], we included HPV31 incident cases in this study; for example, in a prior study, there was approximately 50% cross-protection against HPV31 after vaccination with the bivalent HPV vaccine [42]. In fact, our results show that, independent of HPV type, particularly high-risk controls that had elevated risk of exposure to new HPV infections (but nonetheless did not have detectable HPV16/31 DNA) had higher serum titers of anti-HPV16 antibodies compared to matched cases with incident HPV16/31 infections.

Real-life evidence of HPV vaccine effectiveness has become increasingly evident, especially in females vaccinated before sexual exposure to HPV [43]. In our study, the modeling analysis, adjusting for 4vHPV immunization before initiating sexual activity, showed that cases were 32% less likely to have higher antibody titers than controls (OR = 1.47, *p* = 0.08). Studies conducted in females vaccinated < 15 years of age have shown high effectiveness in reducing HPV vaccine type prevalence [12,44,45,46,47]; moreover, studies in many countries have also demonstrated evidence of cross-protection with a decline in HPV31/45 detection in women vaccinated with the HPV16- and HPV18-containing vaccines [47,48].

Vaccine clinical trial studies on bivalent and quadrivalent HPV vaccines have shown an efficacy of over 80% and 36% in preventing HPV16 or HPV31 persistent infections, respectively [45,49]. In our study, adolescent vaccinated females were followed by HPV testing every six months, but we did not limit the analyses to persistent infections because of sample size issues. In addition, most studies on post-vaccination surveillance focus primarily on HPV detection and CIN2+ detection rather than antibody titer follow-up. Our study shows evidence of a possible link between lower antibody titers and the risk of incident HPV16/31 infections.

In this study, we also evaluated the serum titers in cases at three time points (i.e., pre-, peri-, and post-infection). We observed a stable level of antibody titer over the window of examination. This implies that infection with a vaccine-type HPV did not boost the level of antibody titers acquired after immunization with three doses. Therefore, the higher antibody titers detected in the high-risk control group were unlikely to be related to prior HPV infections. We would argue that we did not detect incident HPV16/31 infections in this group because of their robust antibody titers from vaccination.

There are some limitations to this study. One limitation is the sample size, directly correlated to the low prevalence of HPV16/31 infections in our MSAHC vaccinated cohort. Another limitation is that we did not directly measure neutralizing antibodies, although we believe titers against HPV16 VLPs are a reasonable surrogate. Lastly, we could not absolutely control for exposure to HPV16/31 infections in both groups.

In summary, it is accepted that HPV vaccines substantially modify the incidence and duration of HPV16-induced cervicovaginal neoplasia by restricting virus infections through neutralizing antibodies. We show a prospective association between anti-HPV16 VLP antibody titers and the acquisition of an HPV16/31 infection subsequent to the receipt of three doses of the 4vHPV vaccine. Continued follow-up of vaccinated individuals will be required to determine if waning antibody titers might correlate with an increased risk of HPV infection. However, the elimination of HPV16 and other types by breaking the chain of transmission could effectively wipe out the burden of future infections and related neoplasia and cancers.

## Figures and Tables

**Table 1:** Descriptive statistics of case and control groups.

Variables	Cases (*n* = 43)	Random Controls (*n* = 43)	High-Risk Controls (*n* = 43)	*p*-Value *
**Age at Baseline Mean (years ± SD)**	18.13 ± 1.33	17.85 ± 1.23	17.93 ± 1.19	0.5702
**Age at Coitarche Mean (years ± SD)**	14.84 ± 1.15	14.74 ± 1.38	14.49 ± 1.52	0.4701
**Age at First 4vHPV Dose Mean (years ± SD)**	15.09 ± 2.49	15.19 ± 2.12	15.19 ± 2.25	0.9717
**Race** ***n*** **(%)**				
Hispanic	26 (60.47)	23 (53.49)	24 (55.81)	0.1156 ^&^
African American	17 (39.53)	15 (34.88)	18 (41.86)	
Other	0 (0)	5 (11.63)	1 (2.33)	
**Lifetime Number of Partners ** ***n*** **(%)**				
1	2 (4.65)	7 (16.28)	0 (0)	0.0461 ^&^
2	3 (6.98)	3 (6.98)	1 (2.33)	
3	11 (25.58)	12 (27.91)	9 (20.93)	
4+	27 (62.79)	21 (48.84)	33 (76.74)	
**Number of Past Partners (6 months) ** ***n*** **(%)**				
0	2 (4.65)	3 (6.98)	0 (0)	0.0405 ^&^
1	21 (48.84)	29 (67.44)	19 (44.19)	
2+	20 (46.51)	11 (25.58)	24 (55.81)	
**Chlamydia Trachomatis ** ***n*** **(%)**				
Yes	22 (51.16)	15 (34.88)	35 (81.40)	<0.0001 ^&^
**Any Pregnancy** ***n*** **(%)**				
Yes	18 (41.86)	15 (34.88)	21 (48.84)	0.4260 ^&^
**Emergency Contraception Ever (** ***n*** **(%)**				
Yes	32 (74.42)	25 (58.14)	38 (88.37)	0.0065 ^&^
**Condom Use** ***n*** **(%)**				
Never	17 (39.53)	15 (34.88)	30 (69.77)	0.0022 ^&^
**Anal Sex Ever** ***n*** **(%)**				
Yes	17 (39.53)	12 (27.91)	41 (95.35)	<0.0001 ^&^
**Lifetime Number of Anal Sex Partners ** ***n*** **(%)**				
0	26 (60.47)	31 (72.09)	2 (4.65)	<0.0001 ^&^
1	8 (18.60)	4 (9.30)	20 (46.51)	
2+	9 (20.93)	8 (18.60)	21 (48.84)	
**Risk Score** **Mean ± SD**	7.95 ± 2.53	6.65 ± 2.64	10.58 ± 0.59	<0.0001
**Vaccine Before Coitarche ** ***n*** **(%)**				
**Yes**	19 (44.19)	17 (39.53)	13 (30.23)	0.4008 ^&^
**Follow-up Time (years)** **Median (Min, Max)**	2.17 (0.50, 7.46)	5.88 (0.54, 8.90)	6.15 (0.84, 10.00)	<0.0001 ^#^
**Serum Titer** **Median (Min, Max)**	3872.86 (100.00–68,527.80)	4893.76 (248.90–102,450.03)	7859.28 (588.18–51,658.15)	0.0557 ^#^
**Log Serum Titer** **Mean ± SD**	8.16 ± 1.31	8.54 ± 1.41	8.79 ± 1.13	0.0732
**Log Serum Titer** **Case vs. Random Control**				0.1914 ^
**Log Serum Titer** **Case vs. High-Risk Control**				0.0179 ^

* ANOVA testing; ^&^ Cochran–Mantel–Haenszel test; ^#^, Kruskal–Wallis test; ^, T-test; SD, standard deviation.

**Table 2:** Association between log serum titers and incident cases by HPV type.

	HPV16/31 (*n* = 43)		HPV16 Only (*n* = 26)		HPV31 ONLY (*n* = 17)	
	OR (95% CI) ^§^	*p*-Value	OR (95% CI) ^§^	*p*-Value	OR (95% CI) ^§^	*p*-Value
**Cases vs. Controls**	1.41 (1.01–1.96)	**0.0415**	1.26 (0.82–1.92)	0.2884	1.67 (0.96–2.92)	0.0704
**Cases vs. Random Controls**	1.25 (0.88–1.79)	0.2143	1.11 (0.69–1.77)	0.6746	1.47 (0.81–2.66)	0.2065
**Cases vs. HR Controls**	1.73 (1.03–2.88)	**0.0373**	1.59 (0.80–3.14)	0.1832	1.97 (0.84–4.60)	0.1177

^§^ Odds ratio (OR) (95% confidence intervals, CI) estimated by multivariate conditional logistic regression, adjusting for the variable ‘Vaccine before coitarche’; the fitted regression models probability (relative odds) of a control having a one log unit increase in antibody titers compared to the cases; HPV, human papillomavirus; HR, high-risk.

**Table 3:** Association between log serum titers and incident HPV16/31 cases among participants who completed vaccination before initiating sexual activity.

4vHPV Completed before Coitarche (Case/Control = 19/30)	
OR (95% CI) ^†^	*p*-Value
1.47 (0.95–2.28)	0.0824

^†^ Univariate logistic regression model estimated odds ratio (OR) (95% confidence interval, CI); 4vHPV, quadrivalent HPV6/11/16/18 vaccine; HR, high-risk.

**Table 4:** Descriptive statistics for cases pre-, peri-, and post-infection.

Variables	Pre-Infection (*n* = 43) Mean ± SD	Peri-Infection (*n* = 10) Mean ± SD	Post-Infection (*n* = 32) Mean ± SD
Log Serum Titers	8.16 ± 1.31	8.36 ± 1.30	8.20 ± 0.77
Duration of Time from Last Dose (years)	2.99 ± 2.94	4.40 ± 2.92	6.52 ± 3.04
Serum Collection Time Relative to Infection (years)	−1.41 ± 0.70	0 ± 0	1.51 ± 0.80

SD, standard deviation.
